# Algorithm of Estimation of the Degree of Porosity Homogeneity of Foamed Concretes by Local Volumes by X-ray Computed Tomography Method

**DOI:** 10.3390/ma16083244

**Published:** 2023-04-20

**Authors:** Sergey Osipov, Inga Prischepa

**Affiliations:** 1 National Research Tomsk Polytechnic University, Lenina Av., 30, 634050 Tomsk, Russia; 2 Tomsk State University of Architecture and Building, pl. Solyanaya, 2, 634003 Tomsk, Russia

**Keywords:** foam concrete, porosity, density, X-ray computed tomography, homogeneity of pore distribution

## Abstract

X-ray CT is widely used to study the structure of foam concrete, the quality of which depends on the uniformity in porosity in local volumes (LV) of the samples. The purpose of this work is to substantiate the need to assess the degree of homogeneity of samples in terms of porosity according to LV. To achieve the goal, an appropriate algorithm has been developed and programmed in MathCad. To illustrate the capabilities of the algorithm, foam concrete modified with fly ash and thermally modified peat (TMP) was tested by CT. The information obtained by CT was processed by the proposed algorithm with variations in LV dimensions in order to estimate the distributions of mean values and standard deviations of porosity. Based on the data obtained, a conclusion was made about the high quality of foam concrete with TMP. The proposed algorithm can be used at the stage of improving the technologies for the production of high-quality foam concretes and other porous materials.

## 1. Introduction

In the development of technological progress, much attention is paid to saving energy and resources in building construction and operation as well as the development and introduction of effective wall materials for buildings by maximizing the use of local raw materials and industrial wastes. The thermal properties are used as estimation criteria for the wall material efficiency with the emphasis on standardized strength and durability. The energy efficiency of buildings is provided by cement foam concrete widely used in the construction of building envelopes [1,2,3]. One of the main parameters of foam concrete is porosity, which will significantly affect their other functional characteristics [3,4,5,6,7]: density; thermal conductivity; frost resistance; water absorption; sound permeability; and mechanical properties. With the goal of increasing the competitiveness of foam concrete, great attention is paid to the process management at all operating stages of the wall material, including a scientific rationale for the choice of the initial components such as cement, mineral and organic disperse additives, foaming agent, foam concrete mixture, and molding and curing conditions. The techniques applied allow creating the appropriate porous structure while preparing the foam concrete mixture, which must remain during the product curing. The stability and quality of foam concrete depend on the pore type, size, concentration, and uniform distribution in the bulk material [4,5,6,7]. The average density, strength, heat conductivity, sound protection, and frost resistance of foam concrete depend on the homogeneity of the pore distribution [8,9]. It is thus necessary to develop the scientifically validated principles of homogeneous structure formation during the preparation of the foam concrete mixture and its consistency during transportation, molding, and curing. For this purpose, it is necessary to utilize modern approaches to homogeneity measurements of the pore and structural element distribution to address them in the design of foam concrete with specified properties.

The homogeneity of the pore distribution in products is conventionally determined by experiments [10,11]. The porosity in the local volume is considered a random value. This value distribution is measured by the ratio between the minimum and maximum values of the average porosity of the material (mixture) in the selected local volume. In relation to concrete, this ratio is called the porosity homogeneity coefficient. The measurement accuracy of this parameter depends on the selected volume, pore size distribution, and local volume. It should be noted that the traditional measurement of the porosity homogeneity coefficient of foam concrete is based on the porosity analysis of specimens separated from the test specimen. Such an approach is destructive. The main problems of destructive testing of concrete are low performance, formation of dust particles during grinding, and distortion of the surface structure during mechanical treatment of specimens.

Different types of non-destructive testing are currently widely used in the construction and production of building materials, which provide the quality control of materials and buildings and conformance to the requirements of design and regulatory documentation [12,13,14]. The internal structure of different building materials can be investigated by X-ray computed tomography (XCT) [15,16,17]. The latter is successfully applied for the improvement of the production process of industrial foam concrete, including nontraditional natural raw materials and industrial waste [18,19,20,21]. The analysis of the internal structure of foam concrete and other porous materials considers the pore size as a random value. Random values are characterized by a set of measures of position and scatter. In practice, the measure of position is an average value, whereas the measure of scatter is a standard deviation (STD). The most full characterization of the random value provides the respective density function.

The quality improvement of porous building materials using the thermal conductivity, strength, and lifetime, can be achieved from several requirements for the porous structure of foam concrete. First, the average pore size in the local volume must be significantly lower than the size of this volume. Second, the measure of scatter (STD) must be significantly lower than the average pore size. In practice, the use of these requirements leads to a negligibly low probability of the formation of pores with the size critical to a consumer. Third, the local volume must be minimized, and frontiers of the foam concrete homogeneity must be admissible (consumer-oriented). This requirement can be variable; for example, it can include a range of local volumes and a range of the respective consumer-oriented frontiers of homogeneity. It is worth noting that the porous homogeneity can be evaluated not only by the porosity homogeneity coefficient but also by the average or STD porosity [21,22]. In practice, this is associated with the limited areas of the local physical effect on foam concrete.

The term “local sample” used in the classical approach to the homogeneity calculation of the foam concrete porosity and the term “virtual local volume” used in the algorithms described in [21,22] are nominally close to each other, although the difference between them concerns their physical entity and metrological component. In the first case, local samples are physical bodies, separated from each other and originating from the test specimen. They have a shape of a cube or cylinder and metric sizes corresponding to certain recommendations and standards. In the second case, local volumes are virtual digital images of the initial, nonseparated volumes. The virtual local volume may have the same shape as a local volume, but their sizes can significantly differ. In this case, the size of the local volume and the digital image of test specimens is generally determined by the size of the voxel (a value on a regular grid in three-dimensional space) used in X-ray computed tomography.

There is very little information offered in the literature concerning the algorithms for interpreting the results of foam concrete tests by the X-ray CT method in terms of assessing the degree of homogeneity of test objects in terms of porosity by local volumes, which proves the actuality of the relevant research.

To achieve the goal of the research, it is necessary to solve a number of tasks:

- substantiate the need to assess the degree of homogeneity of foam concrete samples in terms of porosity by local volumes;
- develop an algorithm for assessing the degree of homogeneity of foam concrete samples in terms of porosity parameters by local volumes using the CT method;
- base the experimental tests of several samples of foam concrete using X-ray computed tomography to demonstrate the possibility of using the developed algorithm;
- illustrate the applicability of the developed algorithm for comparing the efficiency of foam concrete production technologies.

## 2. Materials and Methods

### 2.1. Materials

Three types of non-autoclaved foam concrete were used in the XCT investigations of the structure. The specimens represented cubes with a 100 mm edge length. The volume-averaged values of their densities ranged from 630 to 650 kg/m^3^. The spatial structure of foam concrete samples does not depend on the beginning and duration of test intervals.

**Figure 1:**
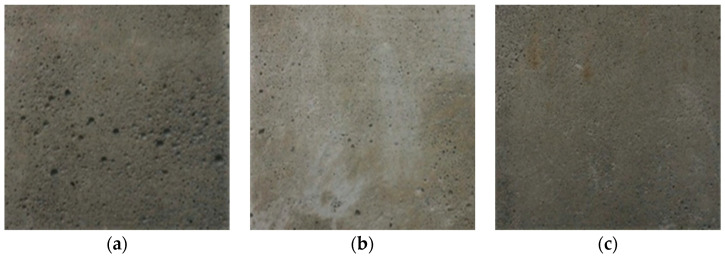
Optical images of non-autoclaved foam concrete specimens [21]: (**a**) Type *1*; (**b**) Type *2*; (**c**) Type *3*.

The first type of foam concrete has no additives and is used for comparison. Scientists in many countries are involved in research concerning the use of industrial wastes in the production of wall materials, for example, bottom ash waste generated by coal power plants [23,24,25]. In the initial dry mixture of the second-type of foam concrete, a part of the cement is replaced by fly ash. In addition to secondary resources, little-used peat is of great interest in the production of building materials [26,27]. The third type of foam concrete is prepared with the addition of thermally modified peat [26]. In Figure 1, the optical images show three types of foam concrete specimens for quality control.

Based on a comparison of the optical images with the foam concrete specimens, we conclude that they are similar. It is, however, not possible to extend this conclusion to their internal structure.

### 2.2. Methods

#### 2.2.1. Used Hardware

**Figure 2:**
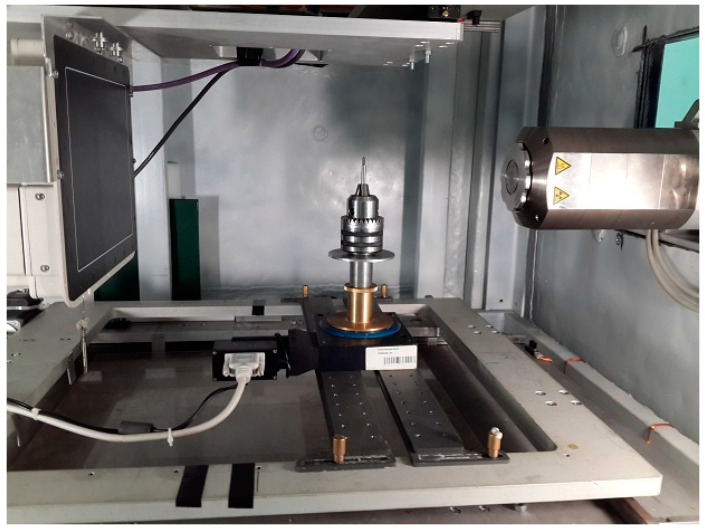
A photograph of XCT system Orel-MT [27].

The XCT system Orel-MT of National Research Tomsk Polytechnic University (Tomsk, Russia) is used for the experimental investigations of foam concrete [28]. Its photograph is presented in Figure 2. The Orel-MT consists of an XWT–160–TC X-ray source (X-RAY WorX GmbH, Garbsen, Germany), a PaxScan 2520V planar detector (Varian Medical Systems, Palo Alto, CA, USA), a rotating table, and a protective box.

Table 1 summarizes the Orel-MT specifications.

The preliminary studies concerned the calculation of the X-ray attenuation by the foam concrete specimens. The analysis of the preliminary results and the data on the internal structure reconstruction proved the sufficiency of the Orel-MT for the purposes of our experiments.

The results of X-ray computed tomography (XCT) are used to evaluate the homogeneity of the pore distribution. The XCT analysis can be reduced to two stages, namely the initial data gathering (full range of projections) and XCT reconstruction.

#### 2.2.2. XCT Projection Formation and Reconstruction

Any foam concrete specimen occupies a certain volume **V**, **V** ⊂ ℜ^3^. The specimen is completely identified, if the porosity *ρ*(*x*,*y*,*z*) and the effective atomic number *Z*(*x*,*y*,*z*) are specified at any point (*x*,*y*,*z*) ∈ **V**. This means that the test specimen is determined by two sets **ρ** and **Z**:

(1) $$\rho =\left\{\rho (x,y,z),\;(x,y,z)\in V\right\},\;Z=\left\{Z(x,y,z),\;(x,y,z)\in V\right\}$$

Concrete is characterized by the uniform distribution of the effective atomic number and nonuniform density distribution:

(2) $$\forall (x,y,z)\in V,\;\rho (x,y,z)=0\vee \rho_{c},\;Z(x,y,z)=Z_{c}$$

where *ρ*_c_ is the density and *Z_c_* is the effective atomic number of hydrated cement. When the density *ρ* is zero at the point (*x*,*y*,z), the latter belongs to a pore, whereas at *ρ*_c_ density, the point (*x*,*y*,z) belongs to hydrated cement.

**Figure 3:**
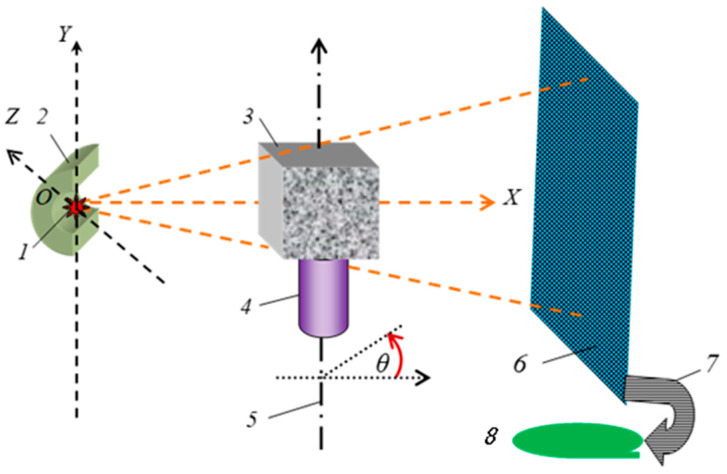
Schematic of projection system geometry in three-dimensional space using non-destructive X-ray imaging: *1*–radiation source, *2*−radiation shielding, *3*–test specimen, *4*−stage of a microscope, *5*−axis of rotation, *6*–planar detector, *7*–information channel, *8*−memory and digital signal processing device.

In Figure 3, one can see a schematic view of the geometry of the projection system in three-dimensional space based on the non-destructive X-ray imaging method for the test specimen [21]. The photon radiation source *1* in radiation shielding *2* irradiates specimen *3* placed on stage *4*, which rotates around axis *5*. Photons suppressed by the specimen are recorded by the planar detector *6* connected to the memory and digital signal processing device *8* via the information channel *7.* Device *8* is equipped with a set of programs for processing the total signals.

Let us connect the origin of the three-dimensional orthogonal coordinate system *OXYZ* with the photon radiation source. The *OX* axis is normal to the frontal surface of the planar detector. The axis of rotation is perpendicular to the *OX* axis and parallel to the *OY* axis. Let the set **S** be a combination of all points of detection with (*y*,*z*) coordinates. The test specimen’s shadow belongs to set **S**.

The XCT formation of the projection system is based on the approach proposed in [19,29]. The X-ray intensity *I* with the maximum energy *E*_max_ is recorded by a scintillation detector of *h_sc_* thickness at the point (*y*,*z*) and is associated with the parameters of the test specimen turned around the axis of rotation to *θ* angle:

(3) $$I(y,z,\theta )=N_{0}(y,z)\int_{0}^{E_{\max}}E_{ab}(E)f(E,E_{\max})\varepsilon (E,h_{sc})e^{-m_{c}(E)\int_{0}^{h(y,z,\theta )}\rho (x,y,z,\theta )dx}dE$$

where *N*_0_(*y*,*z*) is the number of photons bombarding the frontal surface of the planar detector if the specimen is absent; *f*(*E*,*E*_max_) is the energy spectrum of the radiation source; *E_ab_*(*E*) is the average energy of the detected phonon with energy *E*; *ε*(*E*,*h_sc_*) is the energy dependence of phonon detection efficiency; *h*(*y*,*z*,*θ*), *m_c_*(*E*) is the specimen beam thickness in centimeters and energy dependence of the mass attenuation coefficient of phonon radiation; *ρ*(*x*,*y*,*z*,*θ*) is the specimen density at (*x*,*y*,*z*) point at the rotational angle *θ*. The specimen density at a certain point is determined according to (2).

The spectrum of the single-energy gamma-ray source is *f*(*E*) = *δ*(*E* − *E*_0_). In this case, (3) takes the form

(4) $$I(y,z,\theta )=N_{0}(y,z)E_{ab}(E_{0})\varepsilon (E_{0},h_{sc})e^{-m_{c}(E_{0})\int_{0}^{h(y,z,\theta )}\rho (x,y,z,\theta )dx}=I_{0}(y,z)e^{-m_{c}(E_{0})\int_{0}^{h(y,z,\theta )}\rho (x,y,z,\theta )dx}.$$

Equation (4) is then linearized as

(5) $$m_{c}(E_{0})\int_{0}^{h(y,z,\theta )}\rho (x,y,z,\theta )dx=-\ln\frac{I(y,z,\theta )}{I_{0}(y,z)}=P(y,z,\theta )$$

The system of Equation (5) for sets (*y*,*z*,*θ*), where (*y*,*z*) points belong to the set **S** and the rotational angle *θ* is measured within 0–2*π*, can be solved only for unknown variables *ρ*(*x*,*y*,*z*), (*x*,*y*,*z*) ∈ **V** measured during the calibration of *m_c_*(*E*) value. The solution includes a three-dimensional density distribution over the specimen volume. According to Hsieh [30], many highly effective solutions are based on the system of Equation (5) and sufficient data obtained.

Polyenergetic X-ray radiation, that is, the difference between *f*(*E*,*E*_max_) and *δ* functions, leads to a bias of an estimate of the density distribution in the volume **V**. Various methods are used to eliminate or reduce the polyenergetic effect, among which a step wedge calibration [31] and dual-energy method [30,32] are the most applicable. It should be noted that step wedge calibration is efficient for the XCT investigations when the material of the structural fragments slightly differs by the effective atomic number.

The dual energy method allows for identifying the interaction between the X-ray radiation and substance and the distribution of the effective atomic number and the density in the specimen volume. The main limitation of the dual energy method is its low performance stipulated by the necessity of projecting two maximum energies of the X-ray radiation and calibration measurement, which is rather a complicated procedure.

Foam concrete consists of fine sand, cement paste, and a small amount of foaming additive. Hence, the materials of the solid matrix of a certain concrete type do not differ in the effective atomic number. It is worth noting that step wedge calibration is the sole technique to compensate for the polyenergetic effect in the XCT structural analysis of foam concrete.

On the strength of a significant decrease in the systematic shift caused by the polyenergetic X-ray radiation, let us consider its compensation in more detail. For the elimination of the porosity deviations in the parallel-plane specimen intended for calibration, concrete specimens are fabricated with and without a foaming additive, not observing the required conditions for the foam formation. Specimens with steps of *ρH*_1_, *ρH*_2_,…, *ρH_n_* thickness are most easy to produce. After calibration, the discrete function *F*(*ρH*) = *P* is obtained. The function *F*(*ρH*) = *P* is continuous, smooth, and monotonically grows. The inverse function *F*^−1^(*P*) is applied to the obtained mass attenuation coefficient, and the average thickness of the product can be written as *R* = *ρH*.

*Note 1.* The polyenergetic effect compensation by step wedge calibration implies the reduction in the residual error caused by the inhomogeneity of the structural elements relative to the effective atomic number of the material. This is associated with the growth in the maximum X-ray radiation resulting in the dominant contribution of the Compton scattering to the interaction between the X-ray radiation and the substance.

As a result of the polyenergetic effect compensation, the system of Equation (5) transforms into the system of equations, which is of great interest for the structural analysis of the foam concrete specimens. The system of equations after the transformation can be written as

(6) $$\int_{0}^{h(y,z,\theta )}\rho (x,y,z,\theta )dx=R(y,z,\theta ),\;0\leq \theta \leq 2\pi$$

The XCT reconstruction of the density function *ρ*(*x*,*y*,*z*) in the bulk material **V** allows us to solve a series of problems associated with the assessment of the foam concrete properties at a higher level as compared to conventional approaches.

#### 2.2.3. Pore Distribution Homogeneity

*General information*

According to the XCT reconstruction and step wedge calibration, the density distribution is evaluated by the specimen volume **V**. At (*x*,*y*,*z*) ∈ **V** point, the density *ρ* ranges from 0 to *ρ*_max_, the latter refers to cured foam concrete with zero porosity. Consequently, the porosity *η*(*x*,*y*,*z*) can be calculated from

(7) $$\eta (x,y,z)=\left(\rho_{\max}-\rho (x,y,z)\right)/\rho_{\max}$$

and ranges from 0 to 1.

It should be noted that the foam concrete quality is not least determined by the homogeneity of the pore distribution in the local volumes of its specimens. This pore distribution is considered here as a random value. The pore size distribution in the local volumes is rather difficult to study, especially in varying the pore size and comparing this distribution [33,34,35]. As an alternative, a study of certain properties can be performed to provide a homogeneous pore distribution.

Let us consider the calculation algorithm of the pore distribution homogeneity in foam concrete by the average and STD porosity using the XCT-based three-dimensional density distribution. The porosity *η* is a random value, which ranges between 0 and 1. In the local domain **v** ⊂ **V** ⊂ ℜ^3^, it is characterized by the average porosity in the local domain **v** $\bar{\eta_{V}}$ and the STD *ση***_V_**.

The specimen division into local domains **v***_k_*, *k* = 1…*K* provided by the volume **V**, can be realized under the following conditions:

(8) $$\overset{K}{\underset{k=1}{\cup }}v_{k}=V.$$

The way of dividing the volume **V** into local volumes **v***_k_*, *k* = 1…*K*, determines the processing time of the spatial data. Hence, it is advisable to supplement (8) with another condition:

(9) $$v_{i}\cap v_{j}=\emptyset ;\dim v_{k}=k_{0};\;i,j,k=1\ldots K;\;k_{0}\cdot K=\dim V.$$

where Ø is the empty set, dim**A** is the number of voxels forming the set **A**.

Note that the shape of the foam concrete specimens is usually cubic. It is, therefore, logical to consider the local volumes **v** as cubes with edges *b*, *b* < *B*, where *B* is the specimen size. For a convenient functioning of the calculation algorithm of the homogeneity of the local volumes **v**, let us arrange them layer-by-layer, that is, each volume **v** is characterized by indices *l*, *m*, *n*, viz. $l_{0}\times m_{0}\times n_{0}=k_{0},\;l=1\ldots l_{0},\;m=m\ldots m_{0},\;n=1\ldots n_{0}$

(10) $$l_{0}=m_{0}=n_{0}=\sqrt[{3}]{k_{0}}.$$

The size range of the local volumes can decrease from the full volume **V** to a voxel and increase from a voxel to the full volume **V**. Let us consider the algorithm connected with the increase in the local volumes. In practice, the size of the minimum cubic volume **V** is restricted by the minimum edge length *n*_min_ in pixels, whereas the size of the maximum local domain is restricted by the maximum edge length *n*_max_. The latter is measured in pixels, and for the local domain, is determined by its size specified by a consumer and the pixel size, both in millimeters.

*Note 2.* The minimum edge length *n*_min_ must be comparable with the prevailing pore size detected by the structural analysis of foam concrete.

*Calculation algorithm of pore distribution homogeneity*

The calculation algorithm of the pore distribution homogeneity in foam concrete is based on the initial data and their initial transformation unit, a set of limitations, and the flow diagram.

*The initial data and their initial transformation unit*. The initial data processing starts at the input of this unit. These data include the pore distribution **ρ** at the discrete set of points $V_{d}=\left\{(x_{i},y_{j},z_{l}),\;i=1\ldots M,\;j=1\ldots N,\;l=1\ldots L\right\}\subset V$, minimum and maximum edge lengths *n*_min_ and *n*_max_, and the stage number *k_V_* for changing the size of the local volumes.

For convenience, let the set **V***_d_* be a combination of the layers (matrices)

(11) $$V_{d}=\overset{K}{\underset{k=1}{\cup }}V_{d}^{k},\;V_{d}^{k}=\left\{(x_{i},y_{j},z_{k}),\;i=1\ldots N,\;j=1\ldots M\right\}.$$

The spatial pore distribution **ρ** can be written as the general pore distribution by the layers:

(12) $$\rho =\overset{K}{\underset{k=1}{\cup }}\rho^{k},\;\rho^{k}=\left\{\rho_{i\;j}^{k}=\rho (x_{i},y_{j},z_{k}),\;i=1\ldots N,\;j=1\ldots M\right\}.$$

The spatial pore distribution **ρ** transfers to the spatial pore distribution **η**. This transformation occurs according to (7). The spatial pore distribution **η** can be calculated by analogy with (12):

(13) $$\eta =\overset{K}{\underset{k=1}{\cup }}\eta^{k},\;\eta^{k}=\left\{\eta_{i\;j}^{k}=\frac{\rho_{\max}-\rho_{i\;j}^{k}}{\rho_{\max}},\;i=1\ldots N,\;j=1\ldots M\right\},\;\rho_{\max}=\max(\rho ).$$

It should be noted that the value *ρ*_max_ can significantly differ from the value *ρ*_max_ in (7). This is because of the presence of multiple pores in the structural element of foam concrete, which is comparable with the voxel. This factor can be positive or negative, which is determined by the mineral particle bonding around the pores. Information about the true value of the density of cement stone corresponding to a particular sample of foam concrete will allow us to estimate the values of micro-porosity by the minimum local volumes of samples, that is, voxels. For the studied samples of foam concrete, it is not possible to obtain such information.

*Block diagram.* The proposed algorithm includes the following operating procedures, which are given in detail to eliminate errors during the algorithm transformation to the program code.

1. Introduce and set the counter of the algorithm stages to zero, viz. *k_S_* = 0.

2. Increase the stage number, viz. *k_S_* = *k_S_* + 1.

3. Check *k_S_* ≤ *k_V_* condition. If it is satisfied, move to point 4. Otherwise, terminate calculations and move to point 12, where the final conclusions are formed, and recommendations are given for the pore distribution homogeneity.

4. Specify the size of the local volumes for the *k_S_*-th stage, viz. *n*_0_(*k_S_*).

5. Determine the number *k*_0_(*k_S_*) of local volumes at the *k_S_*-th stage:

$$k_{0}(k_{s})=\frac{M\times N\times K}{n_{0}^{3}(k_{s})}.$$

6. Calculate the number of layers *l*_0_, columns *m*_0_, and lines *n*_0_ for the local volumes at the *k_S_*-th stage:

$$l_{0}(k_{s})=m_{0}(k_{s})=n_{0}(k_{s})=\left[\sqrt[{3}]{k_{0}(k_{s})}\right],$$

where [*x*] is the integer part *x*.

7. Introduce and set the counter of the position identification of local volumes to zero at the *k_S_*-th stage, viz. *l* = 0.

7.1. Increase the layer number, viz. *l* = *l* + 1.

7.2. Check *l* ≤ *l*_0_(*k_S_*) condition. If it is satisfied, move to point 7.3. Otherwise, terminate calculations and move to point 8, where the sets of informative parameters $\bar{\eta_{m\;n}^{l}}$ and $\bar{\sigma_{m\;n}^{l}}$ are compared for the *l*-th layer with the specified levels.

7.3. Calculate the layer boundaries *k*_min_, *k*_max_:

$$k_{\min}=(l-1)n_{0}(k_{s})+1,\;k_{\max}=ln_{0}(k_{s}).$$

7.4. Set the integrators of the first two initial porosity moments to zero: *S*_1_*_η_* = 0 and *S*_2*η*_ = 0.

7.5. Introduce and set the counter of the line in the layer to zero at the *k_S_*-th stage, viz. *m* = 0.

7.6. Increase the line number, viz. *m* = *m* + 1.

7.7. Identify the line boundaries *i*_min_, *i*_max_:

$$i_{\min}=(m-1)n_{0}(k_{s})+1,\;j_{\max}=mn_{0}(k_{s}).$$

7.8. Check *m* ≤ *m*_0_(*k_S_*) condition. If it is satisfied, move to point 7.8.1. Otherwise, terminate calculations and move to point 7.1 to consider the next layer.

7.8.1. Introduce and set the counter of the columns in a line to zero at the *k_S_*-th stage, viz. *n* = 0.

7.8.2. Increase the column number, viz. *n* = *n* + 1.

7.8.3. Check *n* ≤ *n*_0_(*k_S_*) condition. If it is satisfied, move to point 7.8.4. Otherwise, terminate calculations and move to point 7.6 to evaluate the selected informative parameters of porosity.

7.8.4. Calculate the column boundaries *j*_min_, *j*_max_:

$$j_{\min}=(n-1)n_{0}(k_{s})+1,\;j_{\max}=nn_{0}(k_{s}).$$

7.8.5. Update the counters of the first two initial porosity moments *S*_1*η*_ and *S*_2*η*_:

$$S_{1\;\eta }=S_{1\;\eta }+\sum_{i=i_{\min}}^{i_{\max}}\sum_{j=j_{\min}}^{j_{\max}}\sum_{k=k_{\min}+1}^{k_{\max}}\eta_{i\;j}^{k},\;S_{2\;\eta }=\sum_{i=i_{\min}}^{i_{\max}}\sum_{j=j_{\min}}^{j_{\max}}\sum_{k=k_{\min}+1}^{k_{\max}}{\left(\eta_{i\;j}^{k}\right)}^{2}.$$

7.8.6. Calculation of the selected average value $\bar{\eta_{m\;n}^{l}}$ and the STD $\bar{\sigma_{m\;n}^{l}}$ of porosity in the local volume matching indices *l*, *m*, *n*:

$$\bar{\eta_{m\;n}^{l}}=\frac{S_{1\;\eta }}{k_{0}(k_{s})},\;\bar{\sigma_{m\;n}^{l}}=\sqrt{S_{2\;\eta }\frac{k_{0}(k_{s})}{k_{0}(k_{s})-1}-{\left(\bar{\eta_{m\;n}^{l}}\;\right)}^{2}}.$$

7.8.7. Move to point 7.8.2.

8. Compare $\bar{\eta_{m\;n}^{l}}$ and $\bar{\sigma_{m\;n}^{l}}$ in the local volumes at the *k_S_*-th stage with the specified levels.

9. Visualization of the spatial distribution of $\bar{\eta_{m\;n}^{l}}$ and $\bar{\sigma_{m\;n}^{l}}$ informative parameters.

10. Move to point 2.

11. Forming a unit of conclusions and recommendations.

*Note 3.* The most complete characteristic of porosity is the sampling density of distribution *f*(*η*). This means that the stage of calculating the informative parameters can be supplemented by the function *f*(*η*) evaluated over the full set of the local volumes.

The flow diagram is the algorithm for calculating the homogeneity of the pore distribution in the foam concrete specimens. This algorithm is easily implemented in high-level programming languages and, with certain memory and speed limitations, in Mathcad.

In conformity with Note *2*, we can use the minimum edge length *n*_min_ = 2000/*d_v_* to reduce the time of additional calculations, that is, the minimum edge length equals the number of pixels in 2 mm. The value *n*_min_ can be increased, if necessary.

Strictly speaking, an important result of using the calculation algorithm, is the condition *n*_lim_ ≥ *n*_min_. Here, *n*_lim_ is the size of the local volume of the foam concrete specimen. Note that the sampling values of the average porosity are close to each other for all local volumes of specimens with a size greater than *n*_lim_; the same is true for the STD porosity deviation and sampling density distribution.

The indicated sampling values of the informative parameters must satisfy the consumer demands. The value *n*_lim_ is the additional parameter describing the foam concrete quality, since the lower the *n*_lim_, the higher the functional properties of foam concrete.

## 3. Results

### 3.1. XCT Visualization of Internal Structure of Foam Concrete Specimens

The operating parameters of the Orel-MT included 160 keV energy of X-ray radiation, −1.7 geometric magnification, and 2 mm copper filter thickness. Geometric magnification here was the ratio between the linear size of the specimen shadow and the linear transverse size of a voxel. The voxel size *d_v_* approached 75 µm. The internal parts of 51.3 × 51.3 × 51.3 mm^3^ specimens were visualized. The number of layers was 684; each layer consisted of 684 × 684 pixels. The denser elements of the reconstructed internal structure were compared with a lighter shade of gray, whereas lower dense elements were compared with a darker shade of gray.

In industrial applications, it is, however, advisable to use the approach described in [21,23,33], where the white color indicates a pore, and the black color indicates the maximum compaction.

**Figure 4:**
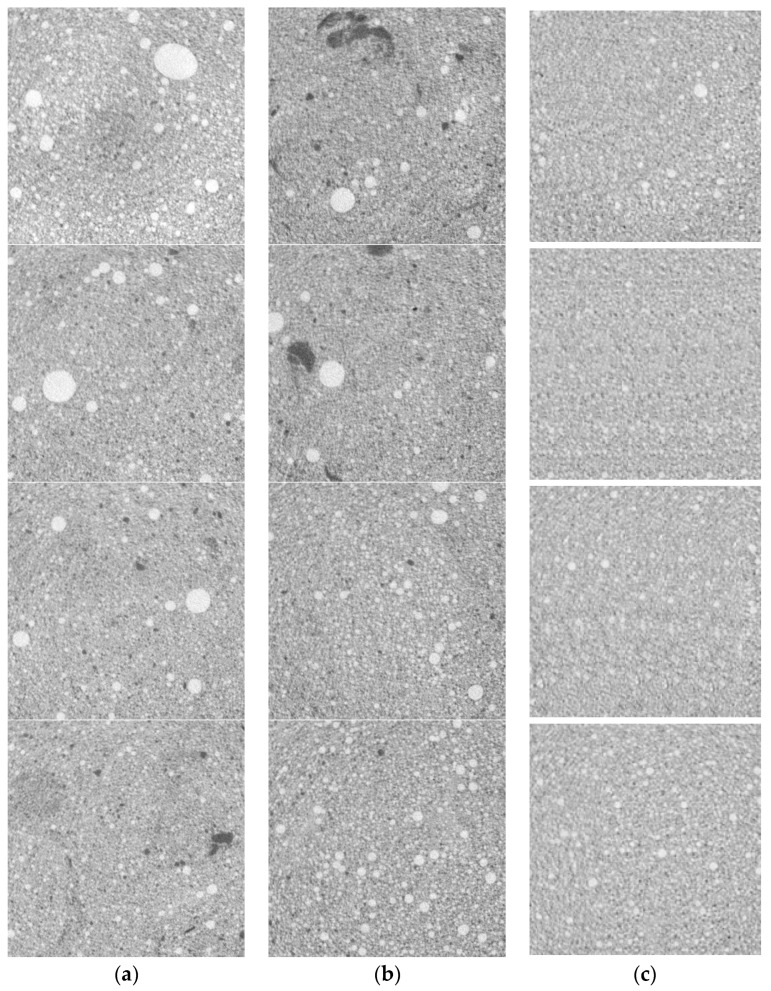
XCT reconstructions of foam concrete structure: (**a**) Type *1*; (**b**) Type *2*; (**c**) Type *3*.

Figure 4 presents the XCT reconstructions of the internal structure of foam concrete using the above-indicated approach to coloring. Each specimen has several images of cross-sections spaced 15 mm apart. The quality of these images is not lower than that obtained in [18,33,34,35]. These images can be used to measure the pore distribution homogeneity in foam concrete for defect identification. It is fair to say that the planar detector of the XCT system used in [35] has better spatial resolution.

The studied samples of foam concrete are close in average value of porosity (0.66–0.74) to the samples from [33,34,35]. As in [33,34,35], the tested test objects contain large spherical pores 2–5 mm in diameter or more, spherical pores of medium size 1–2 mm, finely porous fragments 0.5 mm or less in size, cracks and chains of closely spaced pores, as well as large high-density fragments of indefinite shape and compacted structures.

Recall that the pixel size is close to 75 μm. With this in mind, as a result of a visual comparison of Figure 4a–c, it can be concluded that the sample of the third type has a significantly smaller pore size, better uniformity of pore distribution over the volume, and the absence of large dense fragments larger than 0.3 mm. This implication supports the production technique described in [26] for the non-autoclaved foam concretes with the addition of thermally modified peat. This implication is qualitative and requires quantification, which can be obtained by using the calculation algorithm of the pore distribution homogeneity.

From the analysis of the reconstructed images of the sections of the tested samples of foam concrete of the first (Figure 4a) and, especially, of the second type (Figure 4b), we can conclude that there is a significant difference in the spatial structure of these foam concrete samples by volume. This is the justification for the need to supplement the results of foam concrete testing by X-ray computed tomography with an algorithm for assessing the degree of homogeneity of samples of porosity parameters by local volumes.

### 3.2. Calculation of Pore Distribution Homogeneity

According to Figure 4, the maximum pore size *d*_max_ in all the tree types of non-autoclaved foam concretes is not over 5.5 mm. The calculation algorithm can be approved for local volumes not exceeding 5.5 mm.

Note that the sampling average porosity $\bar{\eta }$ and the STD porosity deviation *ση* in the local volumes are random. It is natural to expect the density function to be significantly narrower than the initial porosity. The quantitative comparison of the pore distribution homogeneity in the local volumes implies the calculation of sampling density distribution of the random values $\bar{\eta }$ and *ση*. The obtained results will improve the production process of foam concrete with higher consumer-oriented properties.

**Figure 5:**
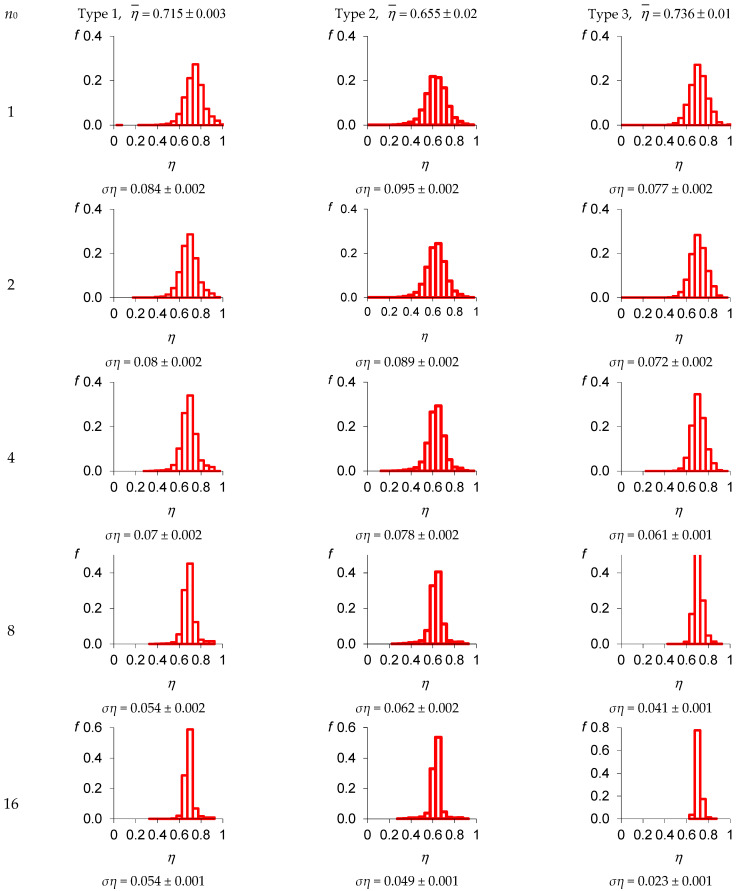
Dependences of porosity informative parameters and local volume size *n*_0_ in pixels.

Based on the proposed algorithm, we developed the calculation procedure in Mathcad to estimate the pore distribution homogeneity in local volumes. The calculation procedure allows formatting images into bmp format images, varying the size of local volumes, estimating sampling average values, STD porosity in local volumes, and sampling density distributions. The experimental results of the informative parameters of porosity are presented in Figure 5, depending on the size of the local volume. The results are given in pixels for the three types of foam concrete.

The calculation procedure was based on the small-to-large volume approach, starting from *n*_0_ = 1 (from a 75 µm volume element of the specimen) to *n*_0_ = 16, which matches 1.2 mm. Seven integrated layers were studied. A slight deviation was observed for the average and STD porosity values for different integrated layers in local volumes.

The analysis of the dependencies in Figure 5 demonstrated the high quality of the foam concrete containing the thermally modified peat additive. This was proven by the higher porosity and its lower STD in local volumes. The narrower density function of the third-type specimen also proved the high concrete quality with increasing size of the local volume.

Local volumes with a size over 1 mm are of great interest for practical applications. Using the developed algorithm, the pore distribution homogeneity is calculated for local volumes 1, 2, 3, and 5 mm in size. The pore distribution homogeneity is calculated by $\bar{\eta }$ and *ση* parameters. Nonnormative compaction and porosity are analyzed for the average porosity in local volumes. In the case of the STD porosity, we study its increase, since its decrease shows the uniform pore distribution and the low pore size in the bulk specimen. The pore size is small if it is much smaller than the local volume.

**Figure 6:**
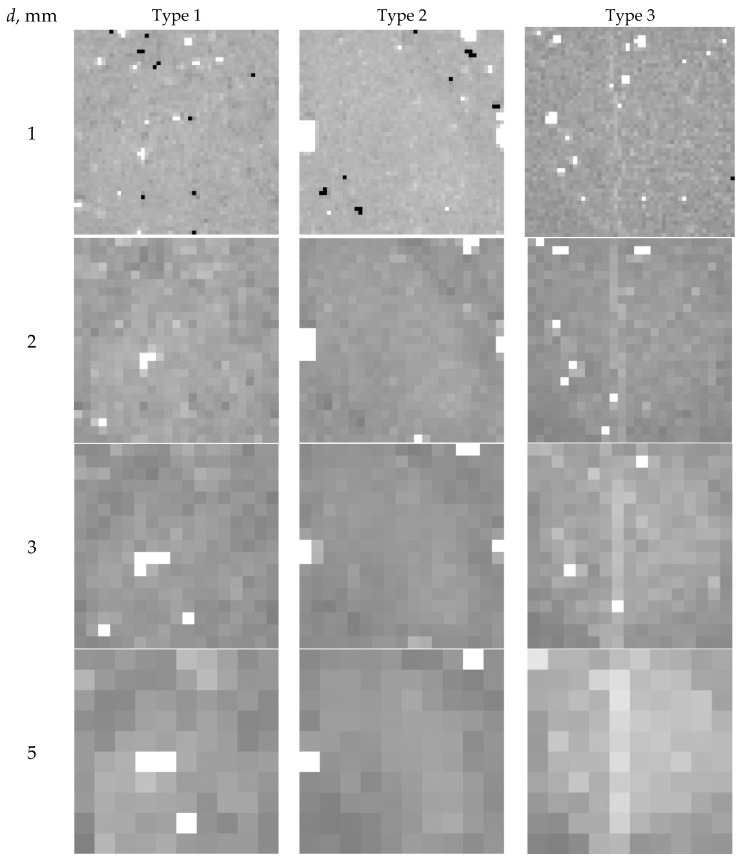
Homogeneity of pore distribution by average porosity $\bar{\eta }$ in local volumes.

The XCT images in Figure 6 show the homogeneity of the pore distribution by the average porosity $\bar{\eta }$ in local volumes with *d* size for all specimens.

The white color in Figure 6 indicates the porosity increase by 3*ση_d_*, whereas the black color indicates the porosity decrease (compaction) by 3*ση_d_*, where *ση_d_* is the STD of porosity in local volumes with *d* size.

**Figure 7:**
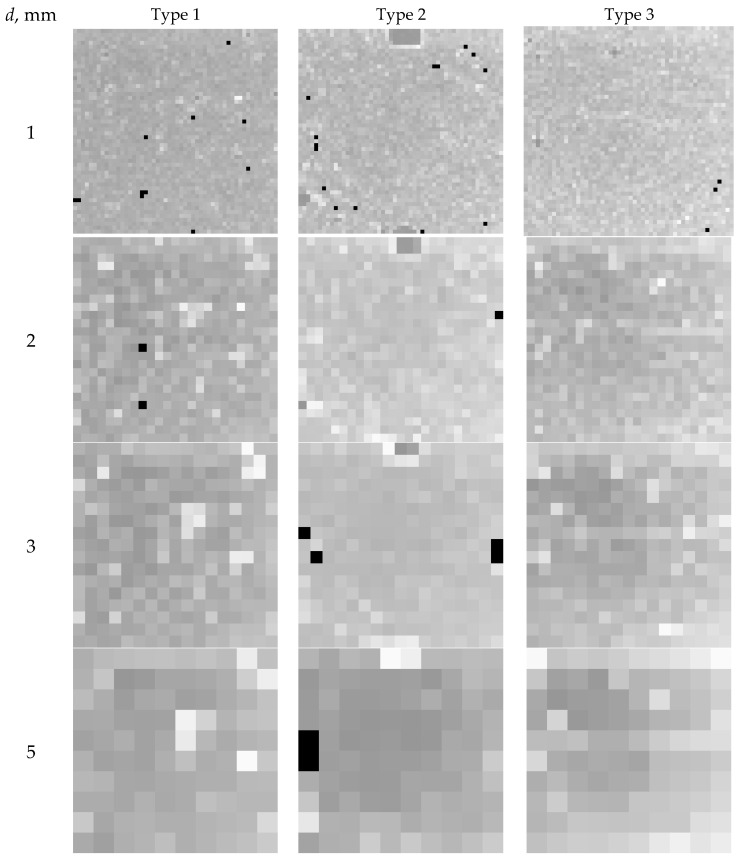
Homogeneity of pore distribution by STD *ση* in local volumes with size *d*, mm.

In Figure 7, the XCT images demonstrate the homogeneity of the pore distribution by the STD of porosity *ση*. The black color indicates a 0.12 *ση* exceedance relative to *ση_d_*.

local volume “Figure 6 and Figure 7 visually illustrate the operation of the proposed algorithm for assessing the degree of porosity homogeneity by local volumes. The mean values and standard deviations of porosity by local volumes are used as parameters characterizing porosity. The work of the proposed algorithm is numerically illustrated by the data shown in Figure 5.

## 4. Discussion

The proposed paper is devoted to substantiating the need and developing an algorithm for assessing the degree of homogeneity of samples of porosity by local volumes using the method of spatial computed tomography. The input of the developed algorithm is the spatial discrete distribution of density **ρ** over the volume of the test volume **V**. As a result of processing the discrete set **ρ** distributions of estimates of the degree of porosity homogeneity by local volumes located in flat parallel layers can be obtained. If researchers need to assess the degree of homogeneity of porosity by local volumes for layers with an arbitrary configuration, then the proposed algorithm can be modified accordingly.

In an explicit form, the proposed algorithm does not allow classifying open and closed pores with sizes comparable to or smaller than voxels. However, testing of foam concrete samples can be supplemented by a study of their moisture absorption throughout the volume. Comparison of the spatial structures of the original and water-saturated samples of foam concretes will allow us to assess the degree of homogeneity of open and closed porosity by local volumes. However, these studies are beyond the scope of this paper.

## 5. Conclusions

The main scientific results of the paper include:

1. Algorithm and program in MathCad for assessing the degree of homogeneity of foam concrete in terms of porosity parameters by local volumes using the X-ray computed tomography method;
2. Demonstration of the possibility of using the developed algorithm based on experimental tests of several samples of foam concrete using X-ray computed tomography;
3. Illustration of the applicability of the developed algorithm for comparing the efficiency of technologies for the production of foam concrete on the example of samples of standard foam concrete and foam concretes modified with fly ash and thermally modified peat;
4. Evidence of the high quality of foam concrete modified with thermally modified peat according to the criterion of a high degree of uniformity in terms of porosity parameters by local volumes, based on high levels of porosity in local volumes, as well as lower values of standard deviations of porosity by local volumes and narrowing of the porosity distribution density as sizes increase local volumes.

The algorithm can be used at the stage of improving production technologies not only for foam concretes, but also for other porous materials, including metal foams.

## Figures and Tables

**Table 1:** XCT system Orel-MT specifications [27].

Spatial resolution	>5 µm
Slice thickness	Variable from 0.5 to 150 mm
Weight capacity	≤20 kg
Pallet dimensions	1150 × 600 × 550 mm
X-ray unit model	XWT 160–TC (X-ray WorX)
Anode voltage	10 to 160 kV
Anode current	0.05–1.0 µA
Focal spot	1.4 µm
Planar detector model	PaxScan 2520 V (Varian)
Pixel size	127 µm
Detector size	193 mm × 242 mm

## Data Availability

Not applicable.
